# Evolving attitudes towards cancer screening: a 2024 update of UK population views

**DOI:** 10.1038/s41416-026-03505-y

**Published:** 2026-06-17

**Authors:** Faye C. Dannhauser, Juliet A. Usher-Smith, Efthalia Massou, Jo Waller, Rebecca A. Dennison

**Affiliations:** 1 Primary Care Unit, Department of Public Health and Primary Care, University of Cambridge, Cambridge, UK; 2 Wolfson Institute of Population Health, Queen Mary University of London, London, UK

**Keywords:** Population screening, Cancer screening, Cancer prevention

## Abstract

**Background:**

This study assessed current population views about cancer screening in light of the Covid-19 pandemic, screening programme changes and new technological advances.

**Methods:**

UK adults (aged ≥18 years), recruited via the online platform, Prolific, completed a questionnaire about their views on cancer screening, using eight questions from a 2012 survey (weighted for age and sex, *n* = 603). We summarised participants’ views, explored associations with participant characteristics using logistic regression and compared 2024 and 2012 responses using Chi-square tests.

**Results:**

Public enthusiasm for cancer screening in the UK remained high with 93% believing routine screening is almost always beneficial, supported by strong beliefs that finding cancer early means treatment saves lives. Awareness of slow-growing cancers was modest at 51% but has increased since 2012. Nevertheless, most participants still wanted testing for slow-growing and untreatable cancer. Some believed they had received too few screening tests, particularly men, people from ethnic minority backgrounds and those with higher educational attainment.

**Conclusions:**

Our study showed that public appetite for screening persists even as awareness of slow-growing cancers increases, reflecting not only possible knowledge gaps but also trust, values and optimism about future treatments. Future research should explore these factors to support truly informed screening decisions.

## Background

Cancer is the second leading cause of death worldwide, accounting for nearly 10 million deaths in 2022 [1]. Globally, cancer incidence rates are projected to increase 55% by 2040 [2]. Screening has the potential to reduce mortality by two mechanisms: by detecting and treating cancer at an early, more treatable, stage and by identifying and treating its precursors [3]. Currently in the UK there are three established population-based national health service (NHS) cancer screening programmes - breast, cervical and colorectal. Additionally, a new targeted screening programme for lung cancer is being implemented over the next five years [4].

Established public attitudes to cancer screening are consistently positive with prior research showing near-uniform enthusiasm for screening across populations [5]. Population surveys have shown that nearly 90% of adult populations in the US (2004) and the UK (2012) agree that “screening is almost always a good idea” [6, 7]. However, since that research was conducted, several important factors may have influenced the public views of cancer screening in the UK, including: changes to cancer screening programmes, the development of new technologies for cancer screening, and the impact of the Covid-19 pandemic.

In 2013, the NHS adopted a new approach to the way information about cancer screening was communicated to the public with more detailed communication regarding the benefits and harms of cancer screening [8]. An example of this was the inclusion of quantitative risk information relating to overdiagnosis in breast cancer screening materials [9]. However, the most recently updated NHS breast screening leaflet (2025) no longer includes these detailed statistics, instead stating broadly that screening may find a cancer that would never have caused harm [10]. This change coincides with a shift towards shorter digital-first communications, including a pilot NHS App booking scheme, and NHS England’s national campaign to increase screening uptake, launched in February 2025 [11]. Various other changes have occurred to the UK cancer screening programmes in the same timeframe. These include the introduction of primary Human Papilloma Virus (HPV) testing for cervical screening in 2019 and the extension of the screening interval from three to five years from 2020 in Scotland, with England and Wales implementing the extension more recently. In bowel screening, changes include decreasing the starting age of screening from 60 to 50 years, using the faecal immunochemical test (FIT) and the dropping of the flexible sigmoidoscopy bowel screening. A targeted lung cancer screening programme for high-risk individuals is also being introduced.

Two further developments since 2012 are particularly relevant to understanding current public views on cancer screening. Firstly, adoption of a personalised risk-based cancer screening approach has been proposed, with a recent meta-analysis showing that increasing screening for high-risk individuals is acceptable to the general public, but that more caution is demonstrated for reducing screening for low-risk individuals [12].

Secondly, advances in genomic medicine have led to the development of Multi-Cancer Early Detection (MCED) tools, which are still in their research phase, but have attracted public interest. Evidence suggests public enthusiasm for MCED tools, primarily due to the simple blood test format and the ability to screen for multiple cancers simultaneously [13, 14]. Both developments have the potential to shift public expectations of what screening programmes should offer, making it timely to reassess population views.

Although surveys have shown that the public consistently expresses positive attitudes towards cancer screening [6, 7] and intentions to participate in screening if invited [15], actual uptake rates are lower. These differences were demonstrated during the Covid-19 pandemic [15]. During the pandemic, worldwide cancer screening rates fell for all cancer types [16]. In the UK, breast cancer screening programmes were particularly affected with coverage in England dropping from 74.1% in 2020 to a low of 64.1% in 2021 [17, 18]. Widespread disruption of cancer screening initiatives led to reduced or suspended services [19, 20]. However, participation in screening remained below pre-pandemic levels for many months after services were reinstated and are only now approaching pre-pandemic levels, with significant regional variation [18, 21, 22]. Although widespread disruption to cancer screening services during the pandemic was a significant factor, attitudinal effects have also played a role. A survey in 2020-21 showed that barriers to screening uptake included fears about Covid-19 infection risk by attending healthcare settings [15].

Given the impact of the Covid-19 pandemic on cancer screening, the evolving landscape of cancer screening technologies, changes to national screening programmes and the complexity of balancing the benefits and harms of cancer screening, it is important to understand how the publics’ views about cancer screening have changed over the last 10 years. In this study we aimed to explore current beliefs about cancer screening among a sample of UK adults, recruited via an online platform, whether these varied across demographic groups, and whether and how they differed from those reported by Waller et al. in 2012 [6]. Whilst not nationally representative, these findings provide contemporary insight into population views on cancer screening at a time of significant change in the UK screening landscape and may help inform future screening communication strategies and research priorities.

## Methods

This study analyses data from a larger survey that included a discrete choice experiment (DCE) exploring public views towards cancer screening programmes with different levels of benefits and harms. Results of the DCE are reported separately. Ethical approval was obtained from the University of Cambridge Humanities and Social Sciences Research Ethics Committee (reference 24.373).

This study is reported in accordance with the Strengthening the Reporting of Observational Studies in Epidemiology (STROBE) guidelines for cross-sectional studies [23, 24].

### Participants and recruitment

A representative sample of the UK population was invited to take part in a survey using Prolific, an online research recruitment platform with a large pool of registered participants (over 200,000 in the UK) who are compensated for completing studies. Prolific enable researchers to recruit samples stratified by age, sex and ethnicity to broadly reflect the UK population, and has been widely used in academic research [25]. All adults were eligible to participate and there were no exclusion criteria. We did not restrict the age to 50–80 years, in part because the parent study (the DCE) examined views towards cancer screening more broadly, and in part to allow exploration of screening views across the full adult age range, including those not yet eligible for screening. For comparability with the 2012 Waller et al. survey, [6] analyses comparing responses between 2024 and 2012 were restricted to participants aged 50–80 years.

Prolific members viewed a brief overview before reading the full participant information sheet and giving online consent if they were interested in taking part. Respondents were able to withdraw at any time without giving a reason by using Prolific’s return submission function. They were compensated £3 for completing the survey.

As this study was primarily descriptive and not specifically designed or powered for between-group comparisons, a post-hoc power calculation was conducted using the pwr package in R for transparency. A two-proportion z-test (pwr.2p2n.test) was applied to estimate statistical power for each of the eight comparisons between the 2024 (50–80 years subset) and 2012 surveys, based on the observed proportions and sample sizes. We note that post-hoc power calculations have recognised limitations and should be interpreted cautiously.

### Survey design

The survey had three components (a) questions about participants’ thoughts about cancer screening, (b) the DCE, which is not included in this publication, and (c) demographics, screening history and cancer worry. For question details see Supplementary Information S1. The eight items used in part (a) were taken from earlier UK and USA surveys [6, 7] to assess views for screening and the demographic questions in part (c) used validated questions where possible [26, 27].

The full survey was discussed and refined with six patient and public representatives through two 1-hour workshops. The public representatives were not asked to suggest changes to the eight items used in part (a) so that we would be able to directly compare participants responses with those of the survey conducted in 2012 [6].

The survey was hosted on the Thiscovery website [28], a research consultancy that was originally developed within the University of Cambridge’s Healthcare Improvement Studies (THIS) Institute as a platform, and now operates independently.

### Analyses

Analyses were carried out using R version 4.5.0. Weights were applied to adjust the sample to be representative of the UK population with respect to age and sex [29]. Summary statistics were used to summarise participants’ characteristics and their responses to the primary questions, and Chi-square statistics were used to test whether screening views differed between the current sample and the 2012 sample [6]. In this latter analysis, participants outside the 50–80 years age range were excluded to reflect eligibility in line with the 2012 sample.

Lastly, adjusted logistic regression was used to explore associations between binary positive endorsements of each screening belief/view, demographic characteristics and previous screening participation. Where continuous or ordinal variables were categorised for regression analyses, the following boundaries were used: age grouped into 18–24, 25–49, 50–59, 60–69, ≥70; ethnicity dichotomised as white or any other ethnicity; educational attainment categorised as GCSEs or none vs A levels or further education vs degree; perceived social position dichotomised as low (1–3) vs middle/high (4–10); perceived likelihood of developing cancer dichotomised as likely (extremely, moderately or slightly likely) vs unlikely (neither likely or unlikely, or extremely, moderately or slightly unlikely); and cancer worry dichotomised as worried (sometimes, often or a lot) vs not worried (rarely or not at all); and participation in cancer screening categorised as yes, no-not invited or no-chosen not to. ‘Prefer not to say’ outcomes were excluded, except for within the explanatory variables where they were treated as missing.

## Results

### Sample characteristics

The survey was conducted on 3 December 2024 with the survey staying open until the target sample size for the full survey (DCE) was attained. 620 participants viewed the information sheet, 4 did not provide consent and 14 withdrew after consenting. After weighting for age and sex, the total sample size was 603. Table 1 summarises respondents’ demographics and experiences of cancer.

**Table 1:** Weighted and unweighted participant responses to questions relating to: (a) demographics and (b) cancer history, screening and worry.

	Unweighted		Weighted	
	*n*	%	*n*	%
Total	602	100	603	100.0
*(a) Participant demographics*				
Sex				
Female	311	51.7	311	51.6
Male	291	48.3	292	48.4
Age category (years)				
18–24	65	10.8	63	10.5
25–49	247	41.0	251	41.6
50–59	144	23.9	104	17.2
60–69	121	20.1	82	13.5
≥70	25	4.2	104	17.2
Broad ethnic group				
Asian	48	8.0	45	7.4
Black	20	3.3	18	2.9
Mixed	10	1.7	12	2.1
White	518	86.0	523	86.8
Other	3	0.5	3	0.5
Prefer not to say	3	0.5	2	0.4
Educational qualifications				
None or GCSEs	82	13.6	86	14.2
A levels or further education	187	31.1	195	32.4
Degree	331	55.0	320	53.2
Prefer not to say	2	0.3	2	0.3
Perceived position on the ‘social ladder’^a^				
1–3 (lowest)	63	10.5	66	10.9
4–6	327	54.3	334	55.4
7–8	196	32.6	189	31.3
9–10 (highest)	7	1.2	6	1.0
Prefer not to say	9	1.5	8	1.3
*(b) Cancer history, screening and worry*				
Personal history of cancer				
Yes	29	4.8	36	6.0
No	569	94.5	564	93.5
Prefer not to say	4	0.7	3	0.5
Family history of cancer				
Yes	256	42.5	261	43.4
No	337	56.0	330	54.7
Prefer not to say	9	1.5	12	1.9
Friend or significant other with cancer				
Yes	373	62.0	399	66.2
No	222	36.9	198	32.8
Prefer not to say	7	1.2	6	1.0
Ever taken part in cancer screening				
Yes	343	57.0	350	58.0
No, have chosen not to have screening	43	7.1	42	7.0
No, have not been invited to have screening	210	34.9	205	34.0
Prefer not to say	6	1.0	6	1.0
Perceived likelihood of developing cancer in 10 years				
Extremely likely	27	4.5	29	4.9
Moderately likely	110	18.3	118	19.5
Slightly likely	164	27.2	159	26.4
Neither likely nor unlikely	149	24.8	143	23.7
Slightly unlikely	29	4.8	33	5.5
Moderately unlikely	55	9.1	51	8.4
Extremely unlikely	34	5.6	36	5.9
Prefer not to say	34	5.6	34	5.7
Worry about cancer				
A lot	22	3.7	22	3.7
Often	46	7.6	49	8.2
Sometimes	214	35.5	221	36.7
Rarely	177	29.4	161	26.8
Not at all	139	23.1	145	24.1
Prefer not to say	4	0.7	3	0.6

Sample weighted for sex and age.

^a^Repeating the question by Prolific when participants registered.

Reflecting the sampling strategy, 52% of participants were female (*n* = 311) and 52% of participants were less than 50 years of age (*n* = 314). Most participants were white (87%) and 13% were from ethnic minority groups (7% Asian, 3% Black, 2% mixed ethnicity and <1% other). Comparison of our total weighted sample with 2021 ONS Census data showed no statistically significant difference in ethnic composition (χ^2^(4) = 8.95, *p* = 0.062, Cramer’s V = 0.061). The largest discrepancy was underrepresentation of the ‘other’ ethnic group (0.5% vs 2.0%), and the modest overrepresentation of white participants. Supplementary information S2. Participants had a higher educational attainment than the population, with over half of participants holding a degree or higher qualifications (53%) [29]. The majority of participants perceived their position on the social ladder (from 1 (low) to 10 (high)) to be within deciles 4–6 (55%) or deciles 7–8 (31%).

Most participants (58%) had taken part in cancer screening, 34% had not been invited for screening whilst 7% had chosen not to have cancer screening. Although only 6% of participants reported a personal history of cancer, many had a family history (43%) and/or knew someone close to them with cancer (66%). Half of participants perceived they were likely to develop cancer within the next 10 years (51%), and 49% worried about it sometimes, often or a lot.

### Overall beliefs

Enthusiasm for screening was extremely high (Table 2) with 93% agreeing that cancer screening is almost always a good idea. Optimism regarding the benefits of screening was reflected by 78% believing that earlier detection means treatment saves lives most or all the time. Just under half (47%) thought early diagnosis means less treatment is needed most or all of the time.

**Table 2:** Views on cancer screening (weighted for age and sex) *n* = 603.

View	Response	*n*	%
View 1 - Do you think routine cancer screening tests for healthy people are almost always a good idea?	Yes No Don’t know/NR	561 19 23	93.0 3.2 3.7
View 2 - How often does finding cancer early mean that treatment saves lives?	All of the time Most of the time Some of the time None of the time Don’t know/NR	37 430 121 1 12	6.2 71.3 20.1 0.2 2.3
View 3 - How often does finding cancer early mean that a person can have less treatment?	All of the time Most of the time Some of the time None of the time Don’t know/NR	27 253 242 18 64	4.5 42.0 40.1 2.9 10.6
View 4 - Have you ever heard of cancers that grow so slowly that they are unlikely to cause you any problems in your lifetime?	Yes No Don’t know/NR	309 235 60	51.2 38.9 9.9
View 5 - Would you want to be tested to see if you had a slow-growing cancer like that?	Yes No Don’t know/NR	451 62 90	74.8 10.3 14.9
View 6 - If there was a kind of cancer for which nothing could be done, would you want to be tested to see if you had it?	Yes No Don’t know/NR	394 96 113	65.3 15.9 18.8
View 7 - In the past, do you think you have had too many screening tests for cancer, too few or about the right number?	Too many About right Too few Never had screening Don’t know/NR	4 159 213 201 26	0.7 26.4 35.5 33.3 4.3
View 8 - Do you feel that someone who does not go for screening is irresponsible?	Yes No Don’t know/NR	320 213 70	53.1 35.3 11.6

*NR* no response.

Although half (51%) had heard of slow growing cancers, a majority (75%) still wanted to be tested for a slow-growing cancer and 65% wanted testing for untreatable cancer. With regards to screening adequacy, just over a quarter (26%) felt that the amount of screening they had received was just right with around a third (35%) believing they had received too few screening tests, and a third (33%) reporting that they had never had screening. Just over half (53%) regarded declining a screening offer as ‘irresponsible’.

### Associations between beliefs and demographics

Table 3 presents variations in beliefs according to demographic factors and cancer screening experiences.

**Table 3:** Associations between binary screening beliefs, and demographic factors, screening experience and cancer worry.

Question (positive belief response)	Routine screening is a good idea (yes)		Finding cancer early means treatment saves lives (most/ all the time)		Finding cancer early means less treatment (most/ all the time)		Heard of slow growing cancer (yes)	
	*n* (%)	OR (95% CI)	*n* (%)	OR (95% CI)	*n* (%)	OR (95% CI)	*n* (%)	OR (95% CI)
Demographic factors								
Age								
18–24	58 (98.3)	Ref 1.00	50 (83.8)	Ref 1.00	33 (60.8)	Ref 1.00	29 (48.0)	Ref 1.00
25–49	234 (96.7)	0.40 (0.05–3.51)	204 (83.6)	1.1 (0.50–2.44)	119 (54.3)	0.70 (0.37–1.33)	110 (49.4)	1.07 (0.58–1.97)
50–59	96 (97.2)	0.56 (0.06–5.50)	77 (75.6)	0.66 (0.29–1.49)	43 (48.4)	0.62 (0.32–1.21)	53 (56.2)	1.61 (0.84–3.11)
60–69	72 (94.7)	0.24 (0.03–2.27)	55 (69.5)	0.50 (0.22–1.13)	30 (40.5)	**0.45 (0.22–0.89)**	46 (66.2)	**2.35 (1.18–4.71)**
70+	100 (96.6)	0.43 (0.03–6.82)	81 (78.2)	0.74 (0.23–2.38)	54 (53.6)	0.84 (0.31–2.26)	70 (73.8)	**3.65 (1.30–10.26)**
Sex								
Female	292 (97.7)	Ref 1.00	240 (79.3)	Ref 1.00	131 (47.0)	Ref 1.00	155 (55.7)	Ref 1.00
Male	269 (96.6)	0.49 (0.18–1.34)	227 (79.3)	0.99 (0.61–1.61)	148 (57.2)	1.50 (0.98–2.29)	154 (58.1)	1.18 (0.78–1.77)
Ethnicity								
White	488 (96.9)	Ref 1.00	399 (78.4)	Ref 1.00	229 (49.5)	Ref 1.00	270 (57.5)	Ref 1.00
Other ethnicity	72 (95.1)	0.49 (0.14–1.67)	66 (85.7)	1.53 (0.78–3.01)	50 (67.2)	**1.89 (1.1–3.21)**	37 (52.3)	1.00 (0.60–1.67)
Education								
GCSEs or none	78 (93.7)	Ref 1.00	71 (83.5)	Ref 1.00	34 (44.3)	Ref 1.00	38 (46.9)	Ref 1.00
A levels or FE	181 (96.9)	2.04 (0.43–9.66)	153 (81.1)	0.71 (0.30–1.71)	91 (51.8)	1.27 (0.59–2.78)	110 (63.6)	**2.72 (1.34–5.50)**
Degree	301 (97.3)	2.92 (0.68–12.59)	242 (77.1)	0.55 (0.24–1.27)	154 (54.3)	1.43 (0.69–2.99)	161 (55.9)	1.88 (0.91–3.87)
Perceived social position								
Middle or high (4–10)	500 (97.6)	Ref 1.00	415 (80.4)	Ref 1.00	252 (52.4)	Ref 1.00	278 (58.8)	Ref 1.00
Low (1–3)	53 (88.1)	**0.17 (0.05–0.54)**	44 (68.8)	**0.46 (0.21–0.98)**	23 (45.7)	0.70 (0.35–1.40)	27 (42.5)	0.51 (0.25–1.02)
History of cancer								
Family history								
No	307 (97.0)	Ref 1.00	264 (82.3)	Ref 1.00	165 (56.6)	Ref 1.00	148 (51.4)	Ref 1.00
Yes	242 (96.1)	0.80 (0.33–1.97)	196 (76.4)	0.76 (0.47–1.23)	111 (47.1)	0.72 (0.46–1.12)	151 (62.0)	1.24 (0.81–1.92)
Friend/significant other history								
No	182 (95.5)	Ref 1.00	151 (79.9)	Ref 1.00	88 (51.7)	Ref 1.00	91 (51.5)	Ref 1.00
Yes	374 (97.1)	1.74 (0.66–4.55)	310 (78.7)	1.02 (0.66–1.57)	189 (51.9)	1.10 (0.75–1.61)	216 (59.3)	1.06 (0.73–1.54)
Screening experience								
Ever had cancer screening								
Yes	334 (98.4)	Ref 1.00	274 (79.6)	Ref 1.00	157 (49.2)	Ref 1.00	197 (63.3)	Ref 1.00
No, not invited	189 (96.4)	0.23 (0.05–1.08)	165 (83.8)	0.94 (0.41–2.13)	104 (57.8)	0.93 (0.52–1.68)	14 (37.8)	0.79 (0.46–1.33)
No, chosen not to	32 (82.3)	**0.06 (0.01–0.30)**	23 (54.9)	**0.22 (0.09–0.52)**	17 (46.5)	0.76 (0.33–1.77)	96 (50.4)	0.45 (0.17–1.20)
Cancer worry								
Perceived likelihood of developing cancer								
Unlikely	241 (95.2)	Ref 1.00	205 (80.8)	Ref 1.00	124 (44.7)	Ref 1.00	120 (50.7)	Ref 1.00
Likely	290 (97.6)	2.29 (0.69–7.58)	240 (78.9)	1.06 (0.60–1.87)	138 (48.8)	0.88 (0.56–1.39)	178 (63.7)	1.46 (0.94–2.27)
Worry about cancer								
Not worried	280 (95.6)	Ref 1.00	239 (80.7)	Ref 1.00	150 (57.1)	Ref 1.00	147 (53.4)	Ref 1.00
Worried	278 (97.7)	1.95 (0.65–5.84)	227 (78.1)	0.85 (0.52–1.36)	129 (47.0)	0.71 (0.47–1.10)	160 (60.1)	1.25 (0.82–1.89)

Question (positive belief response)	Wants testing for slow growing cancer (yes)		Wants testing for untreatable cancer (yes)		Number of screening tests had in the past (too few)		Believes that not going for screening is irresponsible (yes)	
	*n* (%)	OR (95% CI)	*n* (%)	OR (95% CI)	*n* (%)	OR (95% CI)	*n* (%)	OR (95% CI)
Demographic factors								
Age								
18–24	49 (84.7)	Ref 1.00	50 (88.2)	Ref 1.00	18 (86.0)	Ref 1.00	26 (46.3)	Ref 1.00
25–49	200 (91.3)	1.64 (0.66–4.10)	172 (81.7)	0.53 (0.22–1.34)	89 (69.0)	0.46 (0.12–1.77)	88 (41.1)	0.84 (0.46–1.55)
50–59	76 (88.3)	1.20 (0.45–3.21)	67 (79.4)	0.51 (0.20–1.32)	39 (50.8)	**0.21 (0.06–0.81)**	34 (35.8)	0.70 (0.37–1.34)
60–69	47 (73.9)	**0.38 (0.15–0.99)**	46 (70.9)	**0.30 (0.12–0.77)**	24 (35.2)	**0.10 (0.03–0.40)**	28 (39.2)	0.80 (0.41–1.58)
70+	77 (91.6)	1.51 (0.30–7.51)	48 (80.3)	0.55 (0.12–2.40)	43 (52.1)	0.24 (0.05–1.12)	36 (37.9)	0.75 (0.27–2.07)
Sex								
Female	224 (88.5)	Ref 1.00	179 (76.3)	Ref 1.00	91 (43.2)	Ref 1.00	114 (42.6)	Ref 1.00
Male	226 (87.3)	0.84 (0.47–1.50)	214 (84.3)	1.59 (0.94–2.69)	121 (73.2)	**4.17 (2.32–7.50)**	98 (37.2)	0.80 (0.53–1.22)
Ethnicity								
White	389 (88.9)	Ref 1.00	335 (80.6)	Ref 1.00	183 (53.9)	Ref 1.00	182 (39.6)	Ref 1.00
Other ethnicity	31 (41.7)	**0.48 (0.24–0.95)**	57 (79.9)	0.79 (0.42–1.48)	30 (84.5)	**3.38 (1.34–8.50)**	31 (42.8)	1.12 (0.64–1.97)
Education								
GCSEs or none	59 (87.7)	Ref 1.00	50 (71.60)	Ref 1.00	20 (34.2)	Ref 1.00	29 (36.0)	Ref 1.00
A levels or FE	144 (87.9)	0.96 (0.31–3.02)	126 (80.6)	1.51 (0.59–3.88)	67 (58.8)	**2.83 (1.13–7.10)**	75 (45.9)	1.41 (0.67–2.98)
Degree	247 (88.4)	1.05 (0.33–3.32)	217 (82.9)	1.86 (0.87–4.01)	125 (61.6)	**2.67 (1.12–6.33)**	109 (37.9)	1.03 (0.51–2.08)
Perceived social position								
Middle or high (4–10)	395 (88.2)	Ref 1.00	349 (80.9)	Ref 1.00	194 (56.7)	Ref 1.00	188 (40.1)	Ref 1.00
Low (1–3)	48 (85.5)	0.67 (0.23–1.94)	40 (79.2)	0.78 (0.38–1.59)	18 (54.2)	0.83 (0.33–2.09)	22 (39.0)	0.99 (0.48–2.04)
History of cancer								
Family history								
No	252 (88.3)	Ref 1.00	226 (81.9)	Ref 1.00	117 (63.3)	Ref 1.00	122 (42.4)	Ref 1.00
Yes	189 (87.5)	0.95 (0.52–1.71)	159 (78.3)	0.91 (0.51–1.63)	94 (50.2)	0.78 (0.45–1.34)	90 (37.7)	0.86 (0.54–1.37)
Friend/significant other history								
No	153 (88.1)	Ref 1.00	134 (80.8)	Ref 1.00	67 (62.9)	Ref 1.00	63 (38.3)	Ref 1.00
Yes	293 (87.8)	0.97 (0.55–1.70)	256 (80.2)	0.94 (0.58–1.53)	144 (54.4)	0.73 (0.44–1.23)	148 (40.8)	1.16 (0.77–1.74)
Screening experience								
Ever had cancer screening								
Yes	266 (91.2)	Ref 1.00	219 (81.8)	Ref 1.00	144 (49.3)	Ref 1.00	143 (46.0)	Ref 1.00
No, not invited	164 (90.2)	0.50 (0.19–1.31)	18 (50.9)	0.49 (0.23–1.07)	59 (84.4)	1.70 (0.66)	69 (38.1)	**0.48 (0.26–0.88)**
No, chosen not to	17 (49.7)	**0.06 (0.02–0.19)**	151 (83.9)	**0.16 (0.07–0.36)**	7 (64.2)	1.50 (0.45–5.01)	1 (2.0)	**0.02 (0.00–0.16)**
Cancer worry								
Perceived likelihood of developing cancer								
Unlikely	190 (86.1)	Ref 1.00	182 (82.4)	Ref 1.00	82 (56.5)	Ref 1.00	91 (39.9)	Ref 1.00
Likely	237 (89.4)	1.47 (0.78–2.79)	195 (80.0)	0.98 (0.58–1.65)	126 (57.0)	1.70 (0.97–2.97)	112 (39.6)	0.97 (0.63–1.50)
Worry about cancer								
Not worried	213 (83.1)	Ref 1.00	198 (76.4)	Ref 1.00	90 (51.5)	Ref 1.00	106 (38.9)	Ref 1.00
Worried	235 (93.1)	**2.81 (1.42–5.58)**	192 (85.1)	**2.02 (1.13–3.62)**	121 (60.8)	**1.89 (1.08–3.29)**	107 (41.5)	1.08 (0.70–1.68)

Excluding ‘don’t know’ and ‘prefer not to say’ responses; ‘prefer not to say’ characteristics treated as missing therefore sample sizes vary.

All odds ratios are adjusted for age, sex, education and ethnic group.

Bold indicates a significant odds ratio (*p* < 0.05).

*FE* further education, *OR (95% CI)* odds ratio (95% confidence interval), *Ref* reference.

#### Age category

Using the 18–24 years age group as the reference, participants in the 60–69 years age category were less likely to respond positively to the view that finding cancer early means that a person can have less treatment ‘most / all the time’, adjusted odds ratio and 95% Confidence Interval, OR = 0.45 (0.22–0.89). They were more likely to have heard of slow-growing cancers OR = 2.35 (1.18–4.71) but less likely to want testing for slow-growing cancer OR = 0.38 (0.15–0.99), less likely to want testing for untreatable cancer OR = 0.30 (0.12–0.77) and less likely to believe they had received ‘too few’ screening tests OR = 0.10 (0.03–0.40). Compared to the same reference group, participants aged 70 years and over were more likely to have heard about slow-growing cancer OR = 3.65 (1.30–10.26) and those aged 50–59 years less likely to believe they had received ‘too few’ screening tests OR = 0.21 (0.06–0.81).

#### Sex

Male participants were significantly more likely to believe that they had received ‘too few’ screening tests for cancer in the past OR = 4.17 (2.32–7.50) compared to females. No other statistically significant differences were seen in the response to any of the other views.

#### Ethnicity

Participants from minority ethnic backgrounds were more likely to believe that finding cancer early meant having less treatment most or all the time, OR = 1.89 (1.1–3.21) compared to white participants and more likely to believe that they had received ‘too few’ screening tests OR = 3.38 (1.34–8.50). They were less likely to want testing for slow-growing cancer OR = 0.48 (0.24–0.95).

#### Education

Compared to participants with GCSEs or no qualifications (reference group), those with higher educational attainment were more likely to believe that they had received ‘too few’ screening tests for cancer - A levels or further education OR = 2.83 (1.13–7.10) and degree level OR = 2.67 (1.12–6.33). Participants with A levels or further education, but not those with a degree, were more likely to have heard of slow-growing cancers OR = 2.72 (1.34–5.50).

#### Perceived social position

Participants in the lowest perceived social position were less likely to believe that routine screening is a good idea OR = 0.17 (0.05–0.54) and that finding cancer early means treatment saves lives OR = 0.46 (0.21–0.98). Their responses to the other views were not statistically significantly different.

#### History of cancer

Participants with a family member/significant other with a history of cancer did not have statistically significantly different responses to any of the views compared to those participants without this history of cancer.

### Associations between beliefs and previous screening experience

Participants who had chosen not to have screening had statistically significantly different responses to those who had either taken part in screening or had not been invited to screening, being much less positive in their responses. They were less likely to believe that screening was a good idea OR = 0.06 (0.01–0.30) and that finding cancer early saves lives OR = 0.22 (0.09–0.52). They were less likely to want testing for slow-growing cancer OR = 0.06 (0.02–0.19) or for untreatable cancer OR = 0.16 (0.07–0.36). They were also less likely to believe that not going for screening was irresponsible OR = 0.02 (0.00–0.16).

### Associations between beliefs and cancer worry

Perceived likelihood of developing cancer was not significantly associated with any cancer screening views. However, some beliefs varied by frequency of worry: participants who worried about cancer ‘sometimes’, ‘often’ or a ‘lot’ were more likely to want testing for slow-growing cancer OR = 2.81 (1.42–5.58) and untreatable cancer OR = 2.02 (1.13–3.62) and were more likely to believe they had had too few tests for cancer OR = 1.89 (1.08–3.29) compared to participants who ‘rarely’ or ‘not at all’ worried about cancer.

### Comparison of overall beliefs with 2012

Full details regarding demographics for the weighted 2024 sub-group (aged 50–80 years) and 2012 surveys are included in the Supplementary Information S2. Differences were seen in the age distribution between the two surveys with a lower proportion of participants aged 60-69 years and a higher proportion of participants aged 70–80 years in the 2024 compared to 2012 survey, 29% vs 36% and 33% vs 26% respectively. There were no statistically significant differences in ethnicity between the two surveys for the 50–80 years subgroup (χ^2^ (1) = 0.66, *p*-value = 0.416). Direct comparisons could not be made with respect to educational attainment and perceived social position, however the 2024 survey had proportionately fewer responses from those with no qualifications and more from those with qualifications compared to the 2012 survey.

**Fig. 1:**
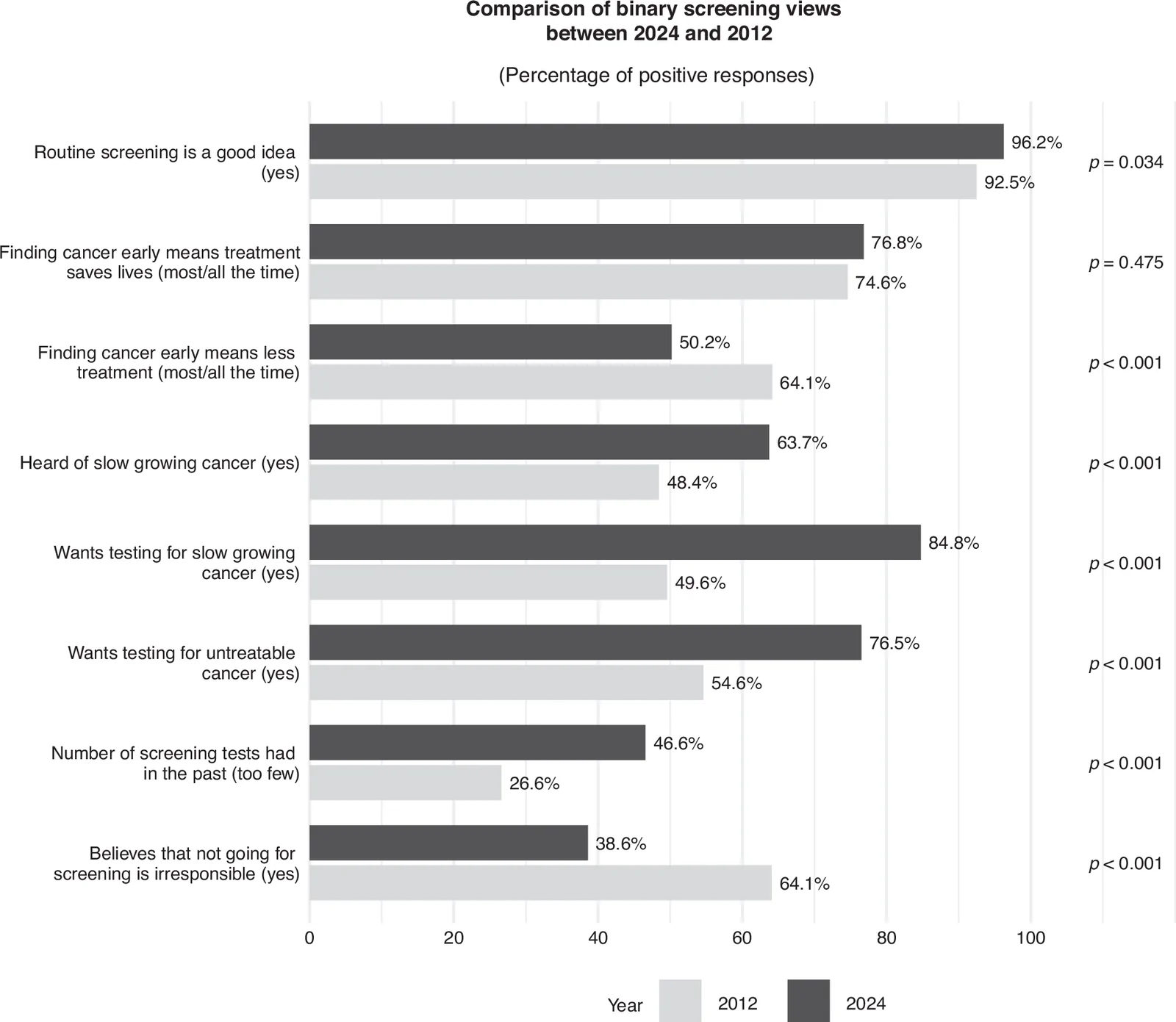
Comparison of binary screening beliefs between 2012 and (aged 50–80 years) 2024 samples. Both samples weighted for age and sex.

Figure 1 compares the 2024 binary positive screening views responses for participants in the 50–80 years age category with the 2012 survey [6]. Statistically significant differences were seen for all the questions (*p* < 0.05) except for the response to finding cancer early means that treatment saves lives all or most of the time.

Post-hoc power calculations indicated that the study had >99% power to detect differences of the observed magnitude for six of the eight beliefs about cancer screening. Power was 71% for the difference in the proportion believing that routine screening is almost always a good idea (2024:96% vs 2012:93%), and this finding should therefore be interpreted with some caution. The non-significant difference in the belief that finding cancer early means that treatment saves lives all or most of the time (2024:77% vs 2012:75%) was consistent with the low estimated power (13%) to detect a difference of this magnitude. Full details are provided in Supplementary Information S3.

Overall, the publics’ preference for cancer screening has increased over the last 10 years. 2024 survey participants in the 50–80 years age-group were more likely to believe that routine cancer screening is almost always a good idea (2024: 96% vs 2012: 93%, *p* = 0.03) but fewer participants believed that finding cancer early meant that a person would have less treatment most or all of the time, (2024: 50.2% vs 2012: 64.1%, *p* < 0.05). There was a greater awareness of slow growing cancers 63.7% vs 48.4% (*p* < 0.05). However, despite this awareness, participants were more likely to want testing for slow-growing cancer 84.8% vs 49.6% (*p* < 0.05) and testing for untreatable cancer 76.5% vs 54.6% (*p* < 0.05). More participants believed they had received ‘too few’ screening tests for cancer, 46.6% vs 26.6% (*p* < 0.05) but fewer participants believed that someone who does not go for screening is irresponsible 38.6% vs 64.1% (*p* < 0.05).

## Discussion

The findings from our study reveal that public enthusiasm for cancer screening in the UK remains consistently high. A substantial proportion of participants (93%) believe that routine cancer screening for healthy individuals is almost always worthwhile. This strong pro-screening sentiment is associated with a widespread belief in the life-saving potential of early detection all or most of the time, reported by 78% of respondents, although only 47% believed that finding cancer early would mean having less treatment all or most of the time.

The positive views towards cancer screening seen in this study exceed the actual cancer screening up-take rates for England in 2023/24 of 71.8% for bowel, 67.5% (ages 25–49 years) and 74.9% (ages 50–64 years) for cervical and 70% for breast cancer [18, 21, 22]. This discrepancy may be explained by several factors. Firstly, individuals more positively disposed towards cancer screening may have been more likely to participate in the survey leading to selection bias. Secondly, significant practical barriers to screening uptake have been reported, such as those seen for cervical cancer screening [30] with common barriers cited including embarrassment, not getting round to it, fear of pain and worry about what might be found. In the real-world setting these factors may play a greater role than in a survey. Thirdly, differences in how participants interpret the question: responses to whether screening tests are almost always a good idea may vary dependent on whether an individual or population basis is considered.

Overall, the positive views about cancer screening are consistent with prior surveys of cancer screening views and those examining public intent to undergo future screening [6, 7, 15]. Some of this enthusiasm may reflect increasing public interest in the development of early detection technologies for cancer such as MCED tools. A recent UK survey (2021) reported that 75% of adults indicated that they would want to be tested every two years when MCED blood tests became available [31]. These tests also appear to be acceptable to the public with a recent US study showing high satisfaction rates for having an MCED test (97.1%) [32].

Only a modest proportion (51%) of participants were aware of slow-growing cancers. However, greater awareness was reported in the over 60’s compared to younger participants and awareness was greater for responses in the 2024 survey (64%) compared to the 2012 survey (48%) for the 50–80 years subset. Despite this growing awareness of slow-growing cancers, a significant proportion of the population still favours testing for cancers that may be slow-growing (75%) or currently untreatable (65%). Since 2012, preferences for such testing have increased by 35% for slow-growing and 22% for untreatable cancer to 85% and 77% respectively. In contrast, there were no significant differences in the belief that finding cancer early means that treatment saves lives between 77% (2024) and 75% (2012). These findings correlate with a 2019 UK survey in which 80% of respondents agreed that they would want cancer diagnosed as early as possible, even if false alarms were raised [33]. Perhaps optimism regarding the potential for future development of cancer treatments explains this discrepancy as recent data indicate that around 65% of adults believe that by 2050 most cancers will either be curable or managed indefinitely with treatment [31].

The finding that awareness of slow-growing cancers has increased since 2012 alongside a desire for testing, raises important questions about the role of risk communication in screening programmes. Hersch et al. conducted a randomised controlled trial of a breast cancer screening decision aid which included quantitative overdiagnosis information. The 2015 paper reported that the intervention increased knowledge and enabled more women to make an informed choice without increasing anxiety or decisional conflict, though fewer women in the intervention group intended to be screened in the short term (74% vs 87%) [34]. Critically, the 2021 long-term follow-up study found that this reduction in intentions did not translate into reduced actual screening participation at two years (55% vs 56%), knowledge gains were sustained and worry about developing breast cancer remained lower in the intervention group throughout [35]. This evidence combined with our finding of a persistent appetite for screening alongside growing awareness of slow-growing cancers, raises the question of whether restoring detailed risk information to NHS screening communications might better support truly informed consent without substantially deterring uptake – a question that warrants further investigation in a UK context.

Compared to 2012, significantly fewer participants believe that not going for screening is irresponsible, 39% (2024) vs 64% (2012). Although participants in the 2024 sample were on average more educated than those in 2012, several other reasons could explain this discrepancy, including greater awareness of screening harms and limitations, increased health autonomy, increased awareness of healthcare system limitations, and the influence of diverse viewpoints on screening on the internet and social media. This finding could also be interpreted optimistically. A reduction in the belief that non-attendance is irresponsible may reflect a welcome shift away from ‘healthism’ ideals. Social norms around screening are important drivers of uptake, and a move towards viewing screening as a genuine choice rather than social duty may ultimately support more truly informed decision-making.

There was also a notable increase in the proportion of participants who believed that they had received too few cancer screening tests 47% (2024) compared to 27% (2012). Men, those with higher educational attainment and those worried about cancer were more likely to respond this way. This perception may have been influenced by socio-political discourse and high-profile media narratives around cancer. The surge in cervical screening rates following Jade Goody’s widely publicised diagnosis in 2008 [36], and more recently, increased advocacy for prostate cancer screening following Sir Chris Hoy’s publicly announced diagnosis in 2024 [37], illustrates the media’s potential to shape public attitudes and policy pressure. Current calls to expand prostate-specific antigen (PSA) testing for high-risk men further reflect this shift towards more targeted screening initiatives [38]. In breast cancer screening, the recently published WISDOM trial in the US provides early evidence that a personalised, risk-based approach may be a safe and effective alternative to standard annual screening [39].

The finding that participants from ethnic minority backgrounds were less likely to want testing for slow-growing cancer, despite being more likely to feel they had received too few screening tests overall, warrants careful interpretation given the small numbers of ethnic minority participants in this sample. A systematic review of barriers to breast and cervical cancer screening among Black, Asian and minority ethnic women in the UK identified five key themes: socio-demographic characteristics; health service delivery; cultural, religious and language factors; gaps in knowledge and awareness; and emotional, sexual and family support [40]. These barriers – particularly gaps in foundational knowledge and awareness and cultural factors – may influence how more complex screening concepts such as testing for slow-growing cancers are perceived and understood.

### Strengths and limitations

We were unable to adjust the sample for socioeconomic status as the measure we used (perceived position on a socioeconomic ladder, which was chosen as participants were already familiar with this question from Prolific’s screening questions) does not equate/map to national indicators. We did not adjust for educational attainment or ethnicity as this was not done in the 2012 analysis [6]. Although the ethnic composition of our weighted sample did not significantly differ from the ONS Census population, the ‘other’ ethnic group remained underrepresented (0.5% vs 2.0%), and the modest overrepresentation of white participants should be considered when interpreting the ethnicity-related findings.

The proportion of participants reporting a family history of cancer (43%) is consistent with the high background prevalence of cancer in the UK, where approximately one in two people will develop cancer in their lifetime. The modestly elevated rate may additionally reflect selection bias, with individuals with a personal connection to cancer potentially more likely to participate in a cancer screening survey. Furthermore, the online recruitment strategy may have also led to selection bias with those with a greater interest in or more positive attitudes towards cancer screening being more inclined to participate. This may partly explain why positive views towards screening in this study exceed actual reported uptake rates.

The higher representation of university educated individuals (53%), and the under representation of those in the lowest socioeconomic group (10%) in our sample may skew findings towards more optimistic views on cancer screening. Other studies have shown individuals from ethnic minority backgrounds and those with lower levels of income and educational attainment are less likely to have positive views about cancer screening [41–45]. The relatively small numbers of ethnic minority participants may have also limited our statistical power to detect meaningful differences in responses across these different groups. As such, caution is warranted in generalising these results to the wider UK population.

Despite these limitations, this study has several notable strengths. Weighting for age and sex helped mitigate some of the social and demographic imbalances. While our weighted sample size, *n* = 603, is smaller than the 2012 comparator study, it provided adequate statistical power for our primary analysis and was sufficient for detecting meaningful differences across key demographic groups. Furthermore, the study’s timing is particularly valuable, capturing public attitudes during a period of significant change in UK cancer screening programmes, providing contemporary data that will be crucial for evaluating the impact of future policy changes.

### Implications and future research

This study highlights overwhelmingly positive views towards cancer screening in the UK. Equally significant is our finding that participants express strong interest in screening for slow-growing or currently untreatable cancers. This apparent paradox warrants deeper investigation. Future research should examine whether this preference reflects insufficient understanding of screening risks and overdiagnosis, genuine optimism about therapeutic advances, or other psychological and social factors that influence screening decisions. Clarifying these motivations is crucial for developing evidence-based communication strategies that support truly informed decision-making, particularly as emerging screening technologies expand both opportunities and complexities in cancer prevention.

## Supplementary information


Supplementary information
STROBE statement


## Data Availability

Data are available in a public, open access repository. All the individual, de-identified participant data collected as part of the study are available on the University of Cambridge repository (https://doi.org/10.17863/CAM.120752). The study protocol, study documents (participant information sheet and consent form), survey and data dictionary are available on the repository. Data are available with no end date without restrictions.
