# Supplementary material for: Evolving attitudes towards cancer screening: a 2024 update of UK population views
Source: Br J Cancer. 2026 Jun 17;135(6):953–63. doi: 10.1038/s41416-026-03505-y (PMC13534454; doi:10.1038/s41416-026-03505-y)
Supplement: Supplementary file 1 [file 41416_2026_3505_MOESM1_ESM.docx]

**(S1) Supplementary Information**

Survey outline – Parts 1 and 4

*[See the full survey outline on the University of Cambridge repository (*[*http://doi.org/10.17863/CAM.120752*](http://doi.org/10.17863/CAM.120752)*) alongside the study protocol, study documents (participant information sheet and consent form), and data.]*

*[Text in blue was not shown to participants. Grey boxes indicate questions posed]*

i) Part 1. Beliefs about cancer screening

**Your thoughts about cancer screening**

Thank you for agreeing to take part in this research.

Before starting the main part of the survey, we want to understand your thoughts and beliefs about cancer screening. Please select the response that best describes your view.

| Do you think routine cancer screening tests for healthy people are almost always a good idea? | - Yes - No - Don’t know |
| --- | --- |
| How often does finding cancer early mean that treatment saves lives? | - None of the time - Some of the time - Most of the time - All the time - Don’t know |
| How often does finding cancer early mean that a person can have less treatment? | - None of the time - Some of the time - Most of the time - All the time - Don’t know |
| Have you ever heard of cancers that grow so slowly that they are unlikely to cause you any problems in your lifetime? | - Yes - No - Don’t know |
| Would you want to be tested to see if you had a slow-growing cancer like that? | - Yes - No - Don’t know |
| If there was a kind of cancer for which nothing could be done, would you want to be tested to see if you had it? | - Yes - No - Don’t know |
| In the past, do you think you have had too many screening tests for cancer, too few or about the right number? | - Too few - About right - Too many - Don’t know |
| Do you feel that someone who does not go for screening is irresponsible? | - Yes - No - Don’t know |

*<next page>*

ii) Part 4. Demographics and cancer worry

**About you**

In this last section, please answer a few questions about yourself. These questions allow us to make sure we include people from a range of backgrounds and see if different groups of people have different views. We will not be able to identify you from your answers.

| How old are you? | - ___ years - Prefer not to say |
| --- | --- |
| What is your biological sex? (A question about gender identity follows) | - Female - Male - Prefer not to say |
| Is the gender you identify with the same as your sex registered at birth? | - Yes - No (please enter your gender identity) - Prefer not to say |
| Which option best describes your ethnic group or background? | - White   - English, Welsh, Scottish, Northern Irish or British   - Irish   - Gypsy or Irish Traveller   - Roma   - Any other White background, please describe__________ - Mixed or Multiple ethnic groups   - White and Black Caribbean   - White and Black African   - White and Asian   - Any other Mixed or Multiple ethnic background, please describe__________ - Asian or Asian British   - Indian   - Pakistani   - Bangladeshi   - Chinese   - Any other Asian background, please describe__________ - Black, Black British, Caribbean or African   - Caribbean   - African, please describe__________   - Any other Black, Black British or Caribbean background, please describe__________ - Other ethnic group   - Arab   - Any other ethnic group, please describe__________ - Prefer not to say |
| What is your highest education level? | - Finished school at or before the age of 15 years - Completed GCSEs, O Levels or equivalent - Completed A Levels or equivalent - Completed further education but not a degree - Completed a Bachelor’s degree - Completed a Master’s degree, PhD or other postgraduate qualification - Other (please describe) |
| Where would you put yourself on the socio-economic ladder? *  *Think of this ladder as representing where people stand in our society. At the top of the ladder (10) are people better off with most education, money and desirable jobs. At the bottom of the ladder (1) are people worse off with least education, money and undesirable jobs. | - 10 – 1 - Prefer not to say |
| Have you ever had cancer? | - Yes - No - Prefer not to say |
| Have your parents or any brothers or sisters ever had cancer? | - Yes - No - Don’t know - Prefer not to say |
| Has anyone close to you (e.g. a partner or close friend) ever had cancer? | - Yes - No - Don’t know - Prefer not to say |
| Have you ever taken part in a cancer screening programme? Examples include:  Bowel cancer screening (poo sample, men and women aged 50 (or 60 depending on region) to 74)  Breast cancer screening (mammogram, women aged 50-71)  Cervical cancer screening (smear test, women aged 25-64) | - Yes - No – I have chosen not to have screening - No – I haven’t been invited to have screening - Prefer not to say |
| How likely do you think is it that you will get cancer at some point in the next 10 years? | - Extremely likely - Moderately likely - Slightly likely - Neither likely nor unlikely - Slightly unlikely - Moderately unlikely - Extremely unlikely - Prefer not to say |
| During the past month, how often have you thought about your own chances of getting cancer? | - Not at all - Rarely - Sometimes - Often - A lot - Prefer not to say |

**References**

1.	Waller J, Osborne K, Wardle J. Enthusiasm for cancer screening in Great Britain: a general population survey. Br J Cancer. 2015 Feb 3;112(3):562–6.

2.	Cancer Research UK, University College London, Kings College London and U of O. Cancer Awareness Measure toolkit version 2.1 Cancer Research UK Cancer Awareness Measure (CAM) Toolkit (version 2.1) [Internet]. 2007. Available from: https://www.cancerresearchuk.org/sites/default/files/health_professional_cancer_awareness_measure_toolkit_version_2.1_09.02.11.pdf

3.	Lerman C, Trock B, Rimer BK, Boyce A, Jepson C, Engstrom PF. Psychological and behavioral implications of abnormal mammograms. Ann Intern Med. 1991 Apr;114(8):657–61.

**(S2) Demographics Table for 2024 vs 2012 Surveys**

i) Weighted and unweighted participant demographics for the 2024 sample (ages 50-80 years only) and 2012 sample.

| **2024** | | | | | **2012** | | | | | **P value for 2024 vs 2012 samples** |
| --- | --- | --- | --- | --- | --- | --- | --- | --- | --- | --- |
|  | **Unweighted** | | **Weighted** | |  | **Unweighted** | | **Weighted** | |  |
|  | **n** | **%** | **n** | **%** |  | **n** | **%** | **n** | **%** |  |
| All | 287 | 100 | 277 | 100 | All | 2024 | 100 | 1895 | 100 |  |
| **Sex** | | | | | **Gender** | | | | | 0.661 |
| Male | 135 | 53.0 | 130 | 47.0 | Male | 951 | 47.0 | 920 | 48.5 |  |
| Female | 152 | 47.0 | 147 | 53.0 | Female | 1073 | 53.0 | 975 | 51.5 |  |
| **Age category** | | | | | **Age group** | | | | | **0.034** |
| 50-59 | 144 | 50.2 | 104 | 37.5 | 50-59 | 658 | 32.5 | 724 | 38.2 |  |
| 60-69 | 121 | 42.2 | 82 | 29.4 | 60-69 | 723 | 35.7 | 674 | 35.6 |  |
| 70-80 | 22 | 7.7 | 92 | 33.1 | 70-80 | 643 | 31.8 | 498 | 26.3 |  |
| **Broad ethnic group** | | | | | **Ethnicity** | | | | | 0.416 |
| White | 262 | 91.3 | 257 | 92.6 | White | 1914 | 94.6 | 1784 | 94.1 |  |
| Black | 7 | 2.4 | 5 | 1.7 | Non-white | 96 | 4.7 | 97 | 5.1 |  |
| Asian | 13 | 4.5 | 9 | 3.4 |  |  |  |  |  |  |
| Mixed | 2 | 0.7 | 4 | 1.6 |  |  |  |  |  |  |
| Other | 0 | 0.0 | 0 | 0 |  |  |  |  |  |  |
| Prefer not to say | 3 | 1.0 | 2 | 0.8 |  |  |  |  |  |  |
| **Educational qualifications** | | | | | **Educational qualifications** | | | | | NA |
| None or GCSEs | 57 | 19.9 | 58 | 21.0 | None | 871 | 43.0 | 738 | 39.0 |  |
| A levels or further education | 79 | 27.5 | 83 | 29.9 | Any educational qualification | 1132 | 57.0 | 1136 | 60.6 |  |
| Degree | 150 | 52.3 | 136 | 48.9 |  |  |  |  |  |  |
| Prefer not to say | 1 | 0.3 | 1 | 0.2 |  |  |  |  |  |  |
| **Ever taken part in cancer screening** | | | | | **Screening history** | | | | | NA |
| Yes | 237 | 82.6 | 235 | 83.3 | ***Colorectal screening (N=1028)*** | | | | |  |
| No, I have chosen not to | 24 | 8.4 | 23 | 8.3 | Eligible and ever attended | 636 | 61.9 | 564 | 62.0 |  |
| No, I have never been invited | 25 | 8.7 | 19 | 6.7 | Eligible and never attended | 392 | 38.1 | 345 | 38.0 |  |
|  |  |  |  |  | ***Breast screening (N=777)*** | | | | |  |
|  |  |  |  |  | Eligible and ever attended | 666 | 85.7 | 635 | 86.2 |  |
|  |  |  |  |  | Eligible and never attended | 111 | 14.2 | 101 | 13.8 |  |

*NA: not appropriate. Bold indicates a significant p-value <0.05.*

ii) Weighted Participant Ethnicity Data: Comparison of 2024 sample (all ages) with ONS Census 2021 data (ages $\geq$ 18 years).

| **Ethnicity** | **2024 sample weighted (n =603)** | **% 2024 sample** | **2012 sample weighted (n= 1895)** | **% 2012 sample** | **ONS Census 2021** |
| --- | --- | --- | --- | --- | --- |
| White | 523 | 86.7% | 1784 | 94.1% | 83.9% |
| Non-white total | 78 | 12.9% | 97 | 5.1% | 16.3% |
| Asian | 45 | 7.5% |  |  | 8.7% |
| Black | 18 | 3.0% |  |  | 3.7% |
| Mixed | 12 | 2.0% |  |  | 1.9% |
| Other | 3 | 0.5% |  |  | 2.0% |
| Prefer not to say | 2 |  |  |  |  |

**(S3) Post-hoc power calculations**

Post-hoc power calculations were conducted for the eight Chi-square comparisons between the 2024 survey (participants aged 50-80 years, with ‘don't know’ responses excluded) and the 2012 Waller et al. survey. Power was estimated using a two-proportion z-test with unequal sample sizes. Cohen's h was used as the effect size measure. A significance level of 0.05 (two-tailed) was applied throughout. Post-hoc power calculations for 2024 vs 2012 comparisons

| **Cancer screening view** | **p 2024** | **n 2024** | **p 2012** | **n 2012** | **Cohen’s h** | **Power** |
| --- | --- | --- | --- | --- | --- | --- |
| V1: Routine screening is almost always a good idea | 0.962 | 266 | 0.925 | 1819 | 0.1658 | 0.714* |
| V2: Early detections saves lives all / most of the time | 0.768 | 272 | 0.746 | 1895 | 0.0518 | 0.126* |
| V3: Early detection means less treatment all / most of the time | 0.502 | 253 | 0.641 | 1896 | 0.2827 | 0.988 |
| V4: Heard of slow-growing cancers | 0.637 | 248 | 0.484 | 1802 | 0.3088 | 0.995 |
| V5: Preference for testing for slow-growing cancer | 0.848 | 223 | 0.496 | 1737 | 0.7771 | 1.000 |
| V6: Preference for testing for untreatable cancer | 0.765 | 213 | 0.546 | 1705 | 0.4670 | 1.000 |
| V7: Believes has had too few screening tests | 0.466 | 219 | 0.266 | 1895 | 0.4186 | 1.000 |
| V8: Believes not going for screening is irresponsible | 0.386 | 254 | 0.641 | 1736 | 0.5153 | 1.000 |

* Power below 0.80 threshold. P = observed proportion; n = sample size (excluding ‘don’t know responses); Cohen’s h = effect size for difference between two proportions.
