# Supplementary material for: Evolving attitudes towards cancer screening: a 2024 update of UK population views
Source: Br J Cancer. 2026 Jun 17;135(6):953–63. doi: 10.1038/s41416-026-03505-y (PMC13534454; doi:10.1038/s41416-026-03505-y)
Supplement: Supplementary file 2 [file 41416_2026_3505_MOESM2_ESM.docx]

STROBE Statement—Checklist for ***Cross-Sectional Studies***

*Manuscript: Evolving attitudes towards cancer screening: a 2024 update of UK population views. Journal: British Journal of Cancer.*

| **Item** | **Item number** | **Recommendation** | **Location in manuscript** | **Reported (Y/N)** |
| --- | --- | --- | --- | --- |
| **Title and abstract** | | | | |
| Title and abstract | 1(a) | Indicate the study's design with a commonly used term in the title or the abstract. | *Title: ‘*Evolving attitudes towards cancer screening: a 2024 update of UK population views.’  *Abstract:* ‘A weighted sample of UK adults (aged $\geq$  18 years), recruited via the online platform Prolific, completed a questionnaire….’ | Y |
|  | 1(b) | Provide in the abstract an informative and balanced summary of what was done and what was found. | Abstract provides background, methods, results and conclusions summarising the cross-sectional survey design, sample, analysis and key findings. | Y |
| **Introduction** | | | | |
| Background/ rationale | 2 | Explain the scientific background and rationale for the investigation being reported. | Background section describes prior surveys, changes to UK screening programmes, development of MCED tools, COVID-19 pandemic impact and rationale for updating 2012 survey. | Y |
| Objectives | 3 | State specific objectives, including any pre-specified hypotheses. | Final paragraph of background: Aims stated as i) describing current beliefs, ii) exploring demographic variation, iii) comparing with 2012 survey. | Y |
| **Methods** | | | | |
| Study design | 4 | Present key elements of study design early in the paper. | Methods, first paragraph: Cross-sectional online survey design described; data drawn from a larger DCE study. | Y |
| Setting | 5 | Describe the setting, locations and relevant dates, including periods of recruitment, exposure, follow-up and data collection. | Methods / Results: survey conducted on 3 December 2024 via Prolific online platform, UK-wide recruitment. | Y |
| Participants | 6(a) | Give the eligibility criteria and the sources and methods of selection of participants. | Methods (Participants and recruitment): All UK adults aged 18 years and above eligible, no exclusion criteria, recruited via Prolific online platform. | Y |
| Variables | 7 | Clearly define all outcomes, exposures, predictors, potential confounders and effect modifiers. Give diagnostic criteria if applicable. | Methods (Survey design and analyses): eight questions about participants’ thoughts about cancer screening described and referenced to S1 supplement. Demographic and cancer history variables defined with validated sources cited. ^1,2^ | Y |
| Data sources | 8 | For each variable of interest, give sources of data and details of methods of assessment (measurement). Describe the comparability of assessment methods if there is more than one group. | Methods: eight items taken verbatim from Waller et al. 2012 ^3^ and Schwartz et al. 2004^4^. Survey hosted on Thiscovery Platform (reference z). Demographic questions used validated instruments^1,2^ . | Y |
| Bias | 9 | Describe any efforts to address potential sources of bias. | Strengths and limitations section: overrepresentation of university-educated and underrepresentation of ethnic minorities and lowest socioeconomic group acknowledged; age/sex weighting applied; caution noted in generalising to the wider UK population. | Y |
| Study size | 10 | Explain how the study size was arrived at. | Methods (Participants and recruitment): Sample size determined by DCE requirements. Post-hoc power analysis was conducted for each of the eight Chi-square comparisons between the 2024 (50-80 years subset) and 2012 surveys based on the observed proportions and sample sizes. | Partial - power analysis added in revision. |
| Quantitative variables | 11 | Explain how quantitative variables were handled in the analyses. If applicable, describe which groupings were chosen and why. | Methods (Analyses): Continuous variables (e.g. perceived social position) categorised as described; age grouped into five categories; ethnicity dichotomised as white vs other ethnic groups; education grouped into three levels. Rationale for categorisation of social position noted (mapped from prolific screening questions). | Y |
| Statistical methods | 12(a) | Describe all statistical methods, including those used to control for confounding. | Methods (Analyses): R version 4.5.0; summary statistics; Chi-squared tests for 2024 vs 2012 comparisons; adjusted logistic regression for demographic associations (adjusted for age, sex, education and ethnic group). | Y |
|  | 12(b) | Describe any methods used to examine subgroups and interactions. | Methods (Analyses): Logistic regression stratified by age, sex, ethnicity, education, perceived social position, cancer history, screening experience and cancer worry. | Y |
|  | 12(c) | Explain how missing data were addressed. | Methods (Analyses): ‘prefer not to say’ outcomes excluded; ‘prefer not to say’ in explanatory variables treated as missing. Sample sizes vary across analyses accordingly. | Y |
|  | 12(d) | If applicable, describe analytical methods taking account of sampling strategy. | Methods (Analyses): Age and sex weighting applied using 2021 ONS Census data^5^. For 2024 vs 2012 comparison, participants outside the 50-80 years age range excluded to match 2012 sample eligibility. | Y |
|  | 12(e) | Describe any sensitivity analyses. | Not formally conducted; Acknowledged as limitation. Weighted versus unweighted sample characteristics presented in Table 1. | Partial |
| **Results** | | | | |
| Participants | 13(a) | Report numbers of individuals at each stage of study, e.g., numbers potentially eligible, examined for eligibility, confirmed eligible, included in the study, completing follow-up and analysed. | Results (Sample characteristics): 620 viewed information sheet, 616 consented and 602 completed survey, weighted n=603. | Y |
|  | 13(b) | Give reasons for non-participation at each stage. | Results (Sample characteristics): 4 participants did not provide consent and 14 withdrew after consenting.  Methods (participants and recruitment): Reasons for non-participation were not collected as participants were not required to provide a reason for withdrawal. | Y |
|  | 13(c) | Consider use of a flow diagram. |  | N/A |
| Descriptive data | 14(a) | Give characteristics of study participants (e.g., demographic, clinical, social) and information on exposures and potential confounders. | Table 1: Full demographic, cancer history and screening experience characteristics presented for weighted and unweighted samples. | Y |
|  | 14(b) | Indicate number of participants with missing data for each variable of interest. | Table 1: Responses to all characteristics included, including ‘prefer not to say responses’ | Y |
| Outcome data | 15 | Report numbers of outcome events or summary measures. | Table 2: frequencies and proportions of all 8 screening belief items.  Table 3: Associations with demographic factors. | Y |
| Main results | 16(a) | Give unadjusted estimates and, if applicable, confounder-adjusted estimates and their precision (e.g., 95% confidence interval). Make clear which confounders were adjusted for and why they were included. | Table 3: adjusted ORs with 95% CIs Presented for all associations. All ORs adjusted for age, sex, education and ethnic group (noted in table footnote). | Y |
|  | 16(b) | Report category boundaries when continuous variables are categorised. | Methods (Analyses): Boundaries stated for categorisation of continuous and ordinal variables used in the regression analyses. | Y |
|  | 16(c) | If relevant, consider translating estimates of relative risk into absolute risk for a meaningful time period. |  | N/A |
| Other analyses | 17 | Report other analyses done, e.g. analyses of subgroups and interactions and sensitivity analyses. | Results: associations with age, sex, ethnicity, education, social position, cancer history, screening experience and cancer worry all reported in text and Table 3. 2024 vs 2012 comparison reported separately. | Y |
| **Discussion** | | | | |
| Key results | 18 | Summarise key results with reference to study objectives. | Discussion, first paragraph: Key results summarised in relation to stated objectives. | Y |
| Limitations | 19 | Discuss limitations of the study, taking into account sources of potential bias or imprecision. Discuss both direction and magnitude of any potential bias. | Strengths and limitations: over-representation of educated participants, under-representation of ethnic minorities and low socioeconomic groups discussed, weighting for age and sex noted as partial mitigation, caution about generalisation to the general population stated. | Y |
| Interpretation | 20 | Give a cautious overall interpretation of results considering objectives, limitations, multiplicity of analyses, results from similar studies and other relevant evidence. | Discussion and implications: Findings interpreted cautiously, cross-referenced to prior surveys^3,4,6^ limitations acknowledged throughout. | Y |
| Generalisability | 21 | Discuss the generalisability, (external validity) of the study results. | Strengths and limitations: Limitations of sample representativeness discussed; caution about generalising to wider UK population stated. | Y |
| **Other information** | | | | |
| Funding | 22 | Give the source of funding and the role of the funders for the present study and, if applicable, for the original study on which the present article is based. | Funding information: NIHR Policy Research Programme (PR-PRU-NIHR206132). Role of funder stated: views are those of the authors and not necessarily those of NIHR or DHSC. | Y |

**References:**

1.	Lerman, C. *et al.* Psychological and behavioural implications of abnormal mammograms. *Annals of Internal Medicine* **114**, 657–61 (1991).

2.	Cancer Research UK. *Cancer Awareness Measure (CAM)*. https://www.cancerresearchuk.org/health-professional/awareness-and-prevention/the-cancer-awareness-measures-cam-plus/archive (2024).

3.	Waller, J., Osborne, K. & Wardle, J. Enthusiasm for cancer screening in Great Britain: a general population survey. *Br J Cancer* **112**, 562–566 (2015).

4.	Schwartz, L. M., Woloshin, S., Fowler, J., Floyd J. & Welch, H. G. Enthusiasm for Cancer Screening in the United States. *JAMA* **291**, 71–78 (2004).

5.	ONS Census 2021. Ethnic group - Census Maps, ONS. https://www.ons.gov.uk/census/maps/choropleth/identity/ethnic-group/ethnic-group-tb-20b/asian-asian-british-or-asian-welsh-bangladeshi.

6.	Wilson, R. *et al.* Intentions to participate in cervical and colorectal cancer screening during the COVID-19 pandemic: A mixed-methods study. *Prev Med* **153**, 106826 (2021).
